# Mapping Sweet Potato Global Research for Sustainable Food Systems: A Bibliometric Perspective

**DOI:** 10.3390/foods15061002

**Published:** 2026-03-12

**Authors:** Miguel Ángel Rincón-Cervera, Sandra López-Arana, Adriano Costa de Camargo, José Luis Guil-Guerrero, Jesús de las Heras-Roger, Carlos Díaz-Romero

**Affiliations:** 1 Institute of Nutrition and Food Technology, University of Chile, Macul, Santiago 7830490, Chile; adrianodecamargo@inta.uchile.cl; 2 Food Technology Division, Agri-Food International Campus of Excellence (ceiA3), University of Almería, 04120 Almería, Spain; jlguil@ual.es; 3 Escuela de Nutrición y Dietética, Facultad de Medicina, Universidad Finis Terrae, Providencia, Santiago 7501015, Chile; slopez@uft.cl; 4 Instituto de Ciencias Aplicadas, Universidad Autónoma de Chile, Santiago 7500910, Chile; 5 Departamento de Ingeniería Química y Tecnología Farmacéutica, Universidad de La Laguna, 38201 San Cristóbal de La Laguna, Spain; jherasro@ull.edu.es (J.d.l.H.-R.); cdiaz@ull.edu.es (C.D.-R.); 6 Cátedra de Agroturismo y Enoturismo de Canarias, Instituto Canario de Calidad Agroalimentaria (ICCA-ULL), 38201 San Cristóbal de La Laguna, Spain

**Keywords:** sweet potato, *Ipomoea batatas*, bibliometric analysis, sustainability, Sustainable Development Goals

## Abstract

Sweet potato (*Ipomoea batatas* L.) has become a relevant crop in global research due to its remarkable resilience to abiotic stress, richness in bioactive compounds, nutritional relevance, and growing importance within sustainability and circular economy frameworks. This study conducted a comprehensive bibliometric analysis of scientific production indexed in Web of Science, Scopus, and PubMed, mapping how research links the crop’s biochemical properties with sustainability-oriented innovation. Literature on bioactive compounds, food waste management, circular economy strategies, and by-product valorization was examined through keyword co-occurrence, authorship networks, citation patterns, and thematic clustering. Results reveal a rapidly expanding research landscape over the past decade, with strong connections between phytochemical composition, health benefits, sustainable cultivation, and industrial applications. Biology, Chemistry, and Food Science emerged as the most interconnected areas. Collaboration networks remain fragmented, and high-income countries achieve disproportionate citation impact, underscoring structural inequalities. Theoretically, this study contributes to understanding how sweet potato research consolidates as a multidisciplinary field aligned with global sustainability goals. Practically, it highlights opportunities to strengthen equitable international collaboration, advance circular economy approaches, and integrate biotechnology with environmental sustainability to support more resilient food systems.

## 1. Introduction

Sweet potato (*Ipomoea batatas* L.) is a globally important staple crop cultivated extensively across Asia, Africa, and the Americas. Although its center of origin is traced to Central America, its agronomic adaptability has enabled widespread cultivation in tropical and subtropical regions. In 2023, global production reached 93.5 million tons, with Asia contributing the largest share (58.2 million tons). China alone accounted for 51.4 million tons, followed by Indonesia (1.56 million tons) and India (1.29 million tons). In Africa, Malawi (8.05 million tons), Tanzania (4.51 million tons), Nigeria (4.08 million tons), Angola (2.0 million tons), and Madagascar (1.3 million tons) were the leading producers, while in the Americas, the United States (1.14 million tons) and Brazil (926,000 tons) represented the principal contributors [1].

Beyond its role as a staple food, sweet potato has attracted growing scientific and industrial interest due to its rich phytochemical profile and remarkable resilience to abiotic stress. In the current global context, the challenge of producing nutritious foods for a rapidly increasing population faces the serious consequences of climate change. Identifying crops capable of withstanding these pressures is critical in terms of food security [2].

Sweet potato is a rich source of diverse bioactive compounds, including phenolic acids, flavonoids, anthocyanins, phytosterols, and carotenoids, with their relative abundance strongly dependent on the variety. Orange-fleshed cultivars are distinguished by their high carotenoid content, whereas purple-fleshed varieties contain greater concentrations of anthocyanins and other phenolic compounds [3,4]. These phytochemicals are associated with antioxidant, anti-inflammatory, antimicrobial, anticancer, hypoglycemic, hepatoprotective, and cardioprotective effects [5,6]. Such properties have driven the growing use of sweet potato-derived ingredients in functional foods, dietary supplements, natural preservatives, and natural colorants [7,8].

Evidence has highlighted the resilience of sweet potato crops to environmental stressors. Plant breeding strategies are actively used to select varieties with enhanced resistance to abiotic challenges [9]. For example, sweet potato demonstrates drought tolerance, with water requirements comparable to maize or sorghum and lower than rice [10], positioning it as a resilient candidate for agricultural systems threatened by global warming [11]. It also tolerates high temperatures, maintaining suitable yields at up to 30 °C. Its salinity tolerance (up to 20 dS m^−1^) surpasses that of major food crops such as rice (3 dS m^−1^), wheat (6 dS m^−1^), and potato (1.7 dS m^−1^). Furthermore, sweet potato exhibits greater ozone tolerance (up to 60 ppb) compared with soybean, rice, wheat (<10 ppb), maize (~25 ppb), and potato (~30 ppb) [10]. These facts position sweet potato as a climate-resilient food source to address the dual challenges of climate resilience and global food security, also offering significant potential for industrial and functional applications.

The valorization of sweet potato by-products aligns with global efforts to reduce food waste and promote circular economy strategies. By-products can be transformed into flour, powders, purees, extracts, or fibers, enabling their use in a wide range of food applications. For example, flour from sweet potato peels can serve as a gluten free alternative in bakery products such as breads, cakes, cookies, and snacks, improving polyphenol content, antioxidant capacity, and dietary fiber levels [12]. It can also function as a binding agent in meat products such as sausages, meatballs, and patties, offering an alternative for populations with gluten intolerance [13]. Dehydrated sweet potato powders, derived from yellow, orange, or purple varieties provide natural colorants and flavoring agents for confectionery, beverages, and dairy products, addressing the growing demand for clean label ingredients. Sweet potato purees and extracts enhance soups, sauces, spreads, and baked goods by contributing flavor, texture, and nutritional value. Extracts enriched with polyphenols and other bioactive compounds are increasingly applied as natural additives, preservatives, and functional ingredients. Beyond food applications, sweet potato phytochemicals demonstrate promising roles in cosmetics, pharmaceuticals, and nutraceuticals, where they are incorporated into creams, lotions, and serums to support skin health [14,15]. Advanced technologies such as microencapsulation, nanoencapsulation, and supercritical CO_2_ processing safeguard compound stability, promote uniform distribution, and optimize delivery in health-oriented products [16].

Valorization pathways extend into non-food sectors as well. In agriculture, sweet potato by-products can be used for vermicomposting or transformed into biofertilizers through solid state fermentation, improving soil quality and crop performance. In bioenergy, anaerobic digestion of sweet potato waste can generate substantial amounts of biogas and biofertilizer, contributing to reductions in CO_2_ emissions, landfill use, and leachate production [17]. Sweet potato residues also represent a promising feedstock for bioethanol production via *Saccharomyces cerevisiae* fermentation, and for biobutanol production through solventogenic *Clostridium* species, offering renewable alternatives with high energy density and compatibility with existing engines. Additionally, starch extracted from sweet potato by-products can be used to produce biodegradable packaging materials [17]. Recent studies have demonstrated that composites incorporating sweet potato by-products exhibit favorable thermal stability and mechanical properties, supporting their potential as sustainable bioplastics aligned with circular economy principles [18,19].

In recent years, reviews have addressed the phytochemical composition, health benefits, and valorization potential of sweet potato [9,10]. Therefore, these topics fall outside the scope of the current work. Existing reviews have addressed specific aspects such as bioactive compounds, functional properties, or sustainability applications, but have not addressed broader thematic trajectories, collaboration networks, or the scientific influence of publications within an integrated sustainability framework. A recent systematic review focused primarily on agronomy, breeding, and abiotic stress tolerance [20], leaving unexplored the convergence of food science and sustainability. This gap is particularly significant because fragmented research efforts can limit the dissemination of innovative methodologies and hinder the development of interdisciplinary strategies aligned with sustainability and circular economy goals.

To address this gap, the present study examines how international scientific production of sweet potato has evolved in relation to bioactive compounds, sustainability, food waste management, circular economy strategies, and by-product valorization. It also analyzes the thematic trajectories, collaboration networks, and citation patterns that characterize this research field, highlighting how these elements reflect structural inequalities.

To achieve these aims, this study conducts a comprehensive bibliometric analysis of international scientific literature on sweet potato (*Ipomoea batatas* L.), with emphasis on bioactive compounds and phytochemicals, sustainability and food waste management, circular economy strategies, and by-product valorization. This approach is aligned with the United Nations Sustainable Development Goals (SDGs) of the 2030 Agenda, which call for the integration of nutrition, technological innovation, and sustainability in transforming food systems. By mapping research trajectories, identifying influential authors and institutions, and visualizing thematic clusters and collaboration networks, this study delivers a comprehensive and integrative perspective on how sweet potato research contributes to global discussions on nutrition, functional foods, industrial innovation, and sustainable resource management. Practically, this work highlights pathways to strengthen equitable international collaboration, foster circular economy approaches, and accelerate knowledge transfer across disciplines. Ultimately, the findings position sweet potato as a strategic crop with growing relevance for health promotion, environmental sustainability, and the development of circular bioeconomy, underscoring its potential to support more resilient and sustainable food systems.

## 2. Methodology

### 2.1. Literature Search

The literature search was carried out in October 2025 across the Web of Science Core Collection, Scopus, and PubMed, following the search strategies outlined in Table 1 and Supplementary Figures S1–S3. Within each database, three different searches were conducted, employing varied keywords combinations.

Original research articles and review papers published in English from 1990 onwards were considered for the bibliometric analysis, while other types of literature (e.g., books, book chapters, conference communications…) as well as works published in languages other than English or prior to 1990 were excluded. Duplicate identification and filtering of eligible records were performed using the Rayyan platform (https://www.rayyan.ai/, Cambridge, MA, USA).

**Figure 1:**
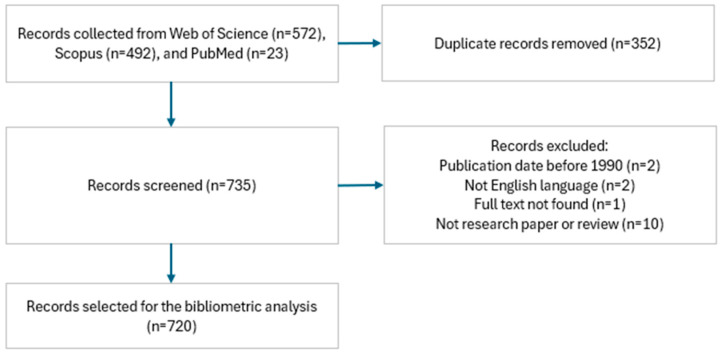
Workflow of the systematic selection of records included in the dataset.

A total of 1087 papers were initially retrieved, which were reduced to 720 after removing duplicates and applying the inclusion and exclusion criteria. The systematic workflow is shown in Figure 1.

### 2.2. Bibliometric Analysis

The resulting records were exported in a compatible format and analyzed using open-source VOSviewer software (version 1.6.20). VOSviewer was selected because it offers a highly accessible, user-friendly environment to explore bibliometric networks without requiring programming expertise [21]. VOSviewer automatically optimized visualization parameters (node size, link strength, and color clustering). Network maps were generated using the full counting method with association strength normalization. The following types of analysis were conducted:

(a) Keywords co-occurrence analysis, to identify thematic connections based on author-declared keywords, grouped into clusters that reflect consolidated research areas. Keywords with at least ten occurrences were included in the map.
(b) Concepts co-occurrence analysis, to uncover thematic structures by automatically extracting and linking terms from titles and abstracts, highlighting how research topics are interconnected in the literature. Terms with a minimum of thirty occurrences in the dataset were considered.
(c) Co-authorship analysis (authors), to map collaboration networks among individual researchers and detect influential partnerships. Authors with at least three co-authored works in the dataset were included in the analysis.
(d) Co-authorship analysis (countries), to visualize international collaboration patterns, showing how countries are connected through joint publications, identifying cooperative clusters, and highlighting countries acting as central hubs of collaboration. Countries with at least five records were considered.
(e) Citation analysis (countries), to assess national influence and visibility within the research field by examining how often works affiliated with a given country are cited by others within the dataset. Countries with at least five records in the dataset were included.
(f) Citation analysis (sources), to illustrate the influence and prestige of scientific journals, highlighting those that serve as central references in the field. Journals with a minimum of five records were considered.

## 3. Results of the Bibliometric Analysis

### 3.1. Longitudinal Trend in Publication Output

**Figure 2:**
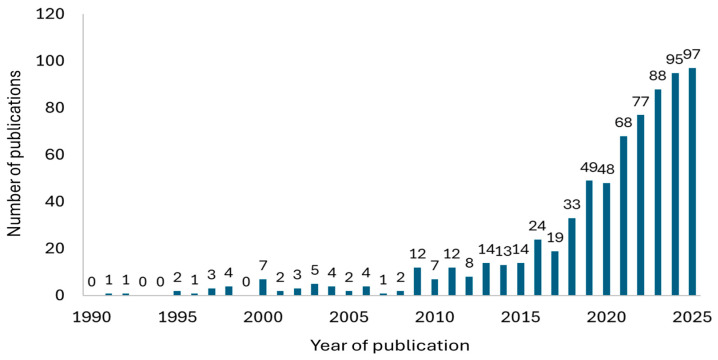
Annual number of publications retrieved from the bibliographic search conducted in the Web of Science, Scopus, and PubMed databases.

The annual number of publications retrieved between 1990 and 2025, following duplicate removal and the application of inclusion and exclusion criteria, is shown in Figure 2. A pronounced increase is evident over the past decade, with 85.0% of the papers (n = 612) published since 2015. This pattern underscores the growing interest of the scientific community in this research field.

### 3.2. Keywords Co-Occurrence Analysis

**Figure 3:**
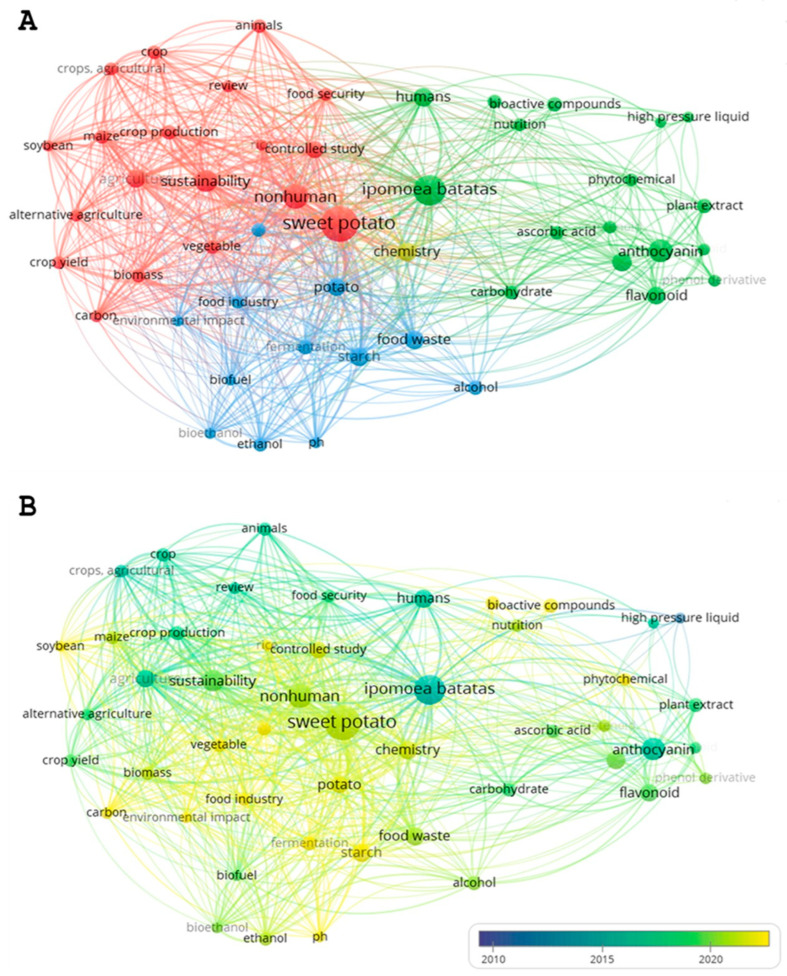
Bibliometric map of keywords co-occurrence analysis, showing thematic clusters (**A**) and their average occurrence over time (**B**).

Keywords are terms explicitly provided by the authors or indexed in the databases, thereby reflecting declared research lines. To simplify the mapping and highlight the most relevant keywords, only keywords with at least 10 occurrences in the dataset were considered for this analysis, yielding 59 out of 3652 terms. The most frequent were *sweet potato* (n = 94), *Ipomoea batatas* (n = 69), *nonhuman* (n = 46), *anthocyanin* (n = 39), and *sustainability* (n = 37) (Figure 3A). VOSviewer grouped keywords into four clusters focused on agricultural production and crop sustainability (red), bioactive compounds and nutritional value of sweet potato (green), biofuels and other industrial applications (blue), and chemistry of sweet potato (yellow). The central terms *sweet potato* and *Ipomoea batatas* are strongly interconnected with related words such as *nutrition*, *phytochemical*, *sustainability*, *alternative agriculture*, *food industry*, *food waste*, *anthocyanin*, *flavonoid*, and *carotenoid*. This way, sweet potato emerges as a bioactive-rich and sustainability-oriented crop. Compounds such as anthocyanins, flavonoids, and carotenoids highlight its nutritional and functional value, while the broader context of sustainability and alternative agriculture underscores its resilience and role in climate-adapted production. Links to the food industry and food waste valorization reveal opportunities for circular economy strategies, turning by-products into valuable ingredients. These dimensions illustrate how sweet potato research integrates health benefits, sustainable cultivation, and industrial applications, reflecting the growing interest of the scientific community in leveraging this crop for both nutritional and environmental impact.

The average occurrence of keywords over time is illustrated in Figure 3B, with the most recent terms (2020–2025) highlighted in yellow or greenish yellow. Keywords such as *sweet potato*, *food industry*, *environmental impact*, *sustainable development*, *biomass*, *phytochemical*, *food waste*, and *bioactive compounds* appear in these colors, reflecting the growing interest in recent years in linking the biochemical composition of sweet potato with industrial applications and sustainability. In contrast, terms such as *crop*, *crop production*, *crop yield*, *food security* or *agriculture* are more prevalent in works published prior to 2020, underscoring a stronger focus on agriculture practices aimed at improving crop yields.

### 3.3. Concepts Co-Occurrence Analysis

**Figure 4:**
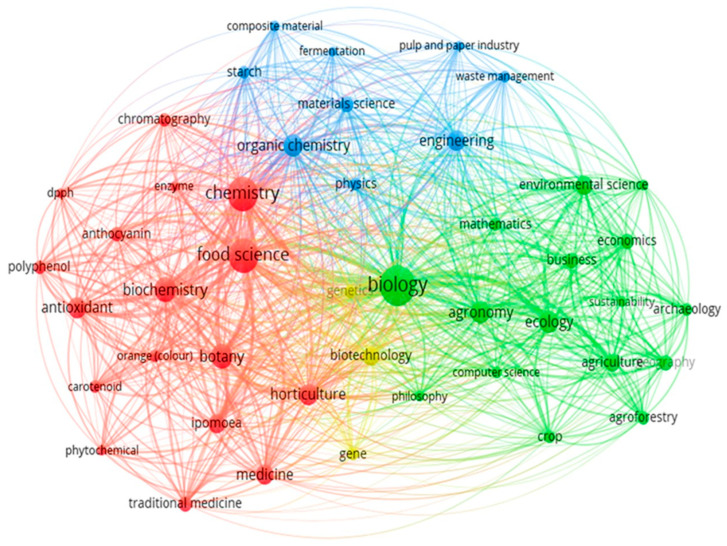
Bibliometric map of concepts co-occurrence analysis, showing thematic clusters.

Concepts are terms automatically generated by the internal algorithm of VOSviewer based on titles and abstracts of publications included in the dataset, using language processing tools. These concepts are then mapped in clusters according to their thematic affinity. The most recurrent concepts in this bibliometric analysis, such as *Biology*, *Chemistry*, and *Food Science*, are represented by larger nodes, reflecting also their strong interconnections (Figure 4).

Concepts included in the red cluster comprises terms related to the chemical composition and bioactive properties of sweet potato, encompassing studies on its chemical and functional profile, and highlighting terms such as *anthocyanin*, *carotenoid*, *antioxidant* and *polyphenol*, with applications in health and food science. The green cluster addresses the sustainable agriculture and socioeconomic context of sweet potato, reflecting agricultural, ecological, and socioeconomic dimensions, and linking sweet potato to agroforestry practices, sustainability, economics, and territorial development. The blue cluster focuses on technological applications and industrial valorization of sweet potato, oriented toward industrial and technological innovation, where starch and other components are utilized in composite materials, fermentation processes, waste management, and applications in the paper and chemical industries. The yellow cluster is related to biotechnology and genetics, focusing on the use of biotechnological tools to enhance the species.

### 3.4. Co-Authorship Analysis (Authors)

**Figure 5:**
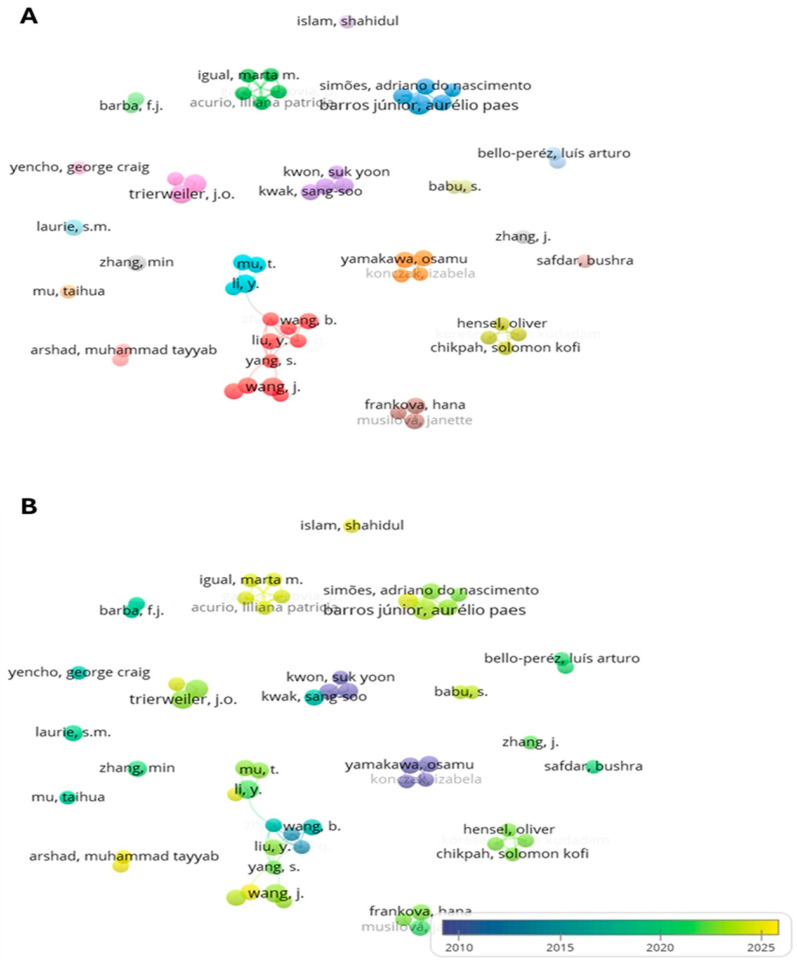
Bibliometric map of co-authorship analysis, showing collaborative networks among authors (**A**) and their average publication dates over time (**B**).

The co-authorship map illustrates collaboration networks among researchers in scientific publications, considering those who have coauthored at least three papers (Figure 5A). Of the 3676 authors identified, 57 meet this threshold. The map reveals several consolidated networks, while many authors remain isolated, indicating that research in the fields addressed by this review may be relatively fragmented, as suggested by the visual patterns observed. Authors within the larger networks are represented by bigger nodes, highlighting the positive influence of collaboration on scientific productivity. However, in general, these networks are not interconnected, which may limit the diffusion of innovative approaches, as novel research tends to circulate within closer circles rather than across broader communities.

The temporal overlay shows how research has accelerated in recent years (Figure 5B). The predominance of green and yellow nodes indicate that many authors have published within the last decade, particularly those belonging to the most productive networks, such as Barros Junior, A.P., and Trierweiler, J.O. This surge coincides with the expansion of sustainability-oriented research, probably driven by external factors such as policy agendas and industrial demands.

While the presence of consolidated groups demonstrates the benefits of sustained partnerships, the lack of interconnection across clusters points to untapped opportunities for collaborative research. Strengthening scientific cooperation could foster interdisciplinary innovation and accelerate the knowledge transference into practice.

### 3.5. Co-Authorship Analysis (Countries)

**Figure 6:**
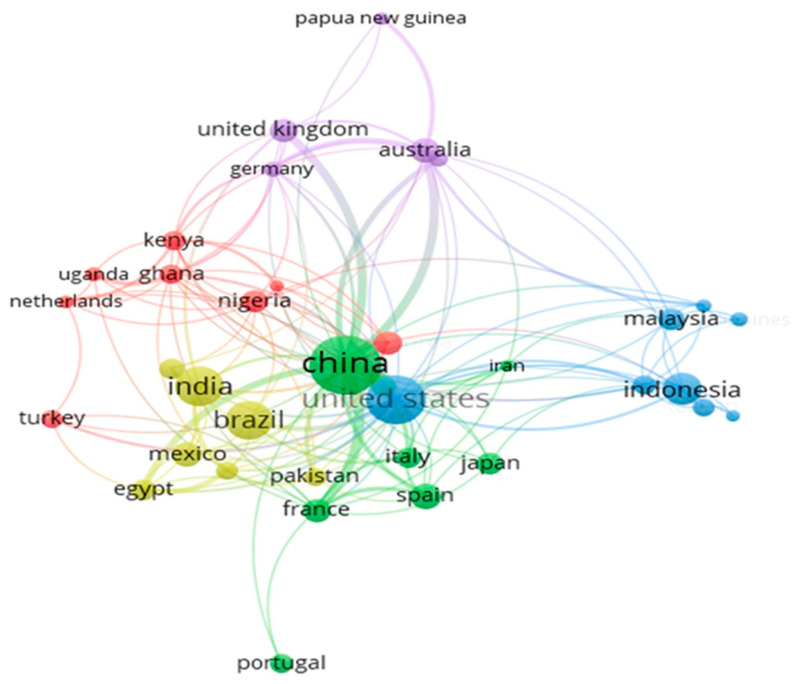
Bibliometric map of international collaborative research networks.

International cooperative networks by country are shown in Figure 6. In total, 36 out of 93 total countries met the requirement of having a minimum of five publications in the dataset to be included in the analysis, with China, the United States, India, Brazil, and Indonesia being countries with the highest number of publications (107, 73, 49, 47 and 33 works, respectively). While the network of China and the United States appear more internationally diversified, those of India, Brazil and Indonesia are comparatively limited. China demonstrates strong cooperation links (indicated by thicker connections in the map) with the United States, the United Kingdom, Australia, France, and Egypt, while the United States shows stronger cooperation with China.

### 3.6. Citation Analysis (Countries)

**Figure 7:**
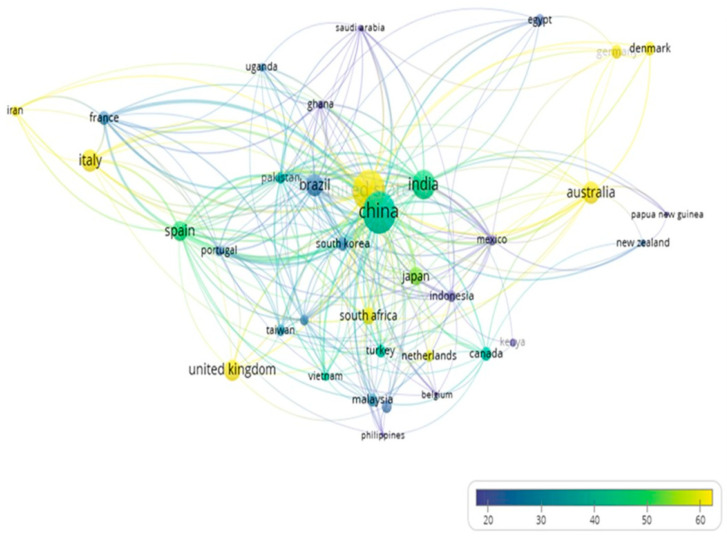
Overlay visualization of the average number of paper citations across countries.

An overlay visualization of the average citations per paper by country is shown in Figure 7. Countries such as the United States, United Kingdom, the Netherlands, South Africa, Iran, Italy, Australia, Germany, and Denmark are represented by yellow nodes, indicating a higher average citation rate. The United States stands out not only for its high publication output but also for its strong citation impact and extensive international collaboration, confirming its central role in the hub. Notably, countries such as the Netherlands, Italy, Iran, Denmark, and Germany, despite having fewer publications, exhibit higher average citation rates than more prolific contributors such as China or India (depicted in green).

### 3.7. Citation Analysis (Sources)

**Figure 8:**
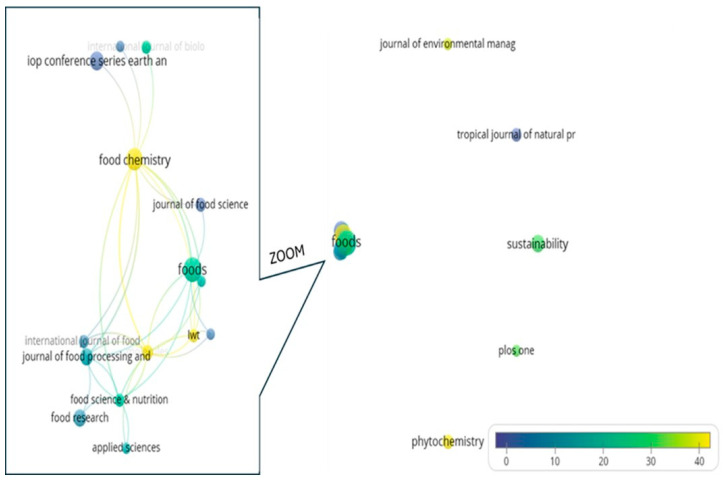
Overlay visualization of the citation analysis of the most representative journals within the dataset.

The influence and impact of scientific journals were assessed based on the number of citations they received from other journals within the dataset. To ensure representativeness, only journals with at least five documents were considered, resulting in 20 out of 376 journals meeting this threshold. Five journals (*Journal of Environmental Management*, *Tropical Journal of Natural Product Research*, *Sustainability*, *Plos One*, and *Phytochemistry*) appear as isolated nodes, while the remaining 15 journals exhibit interconnections (Figure 8). Among these, *Foods* and *Food Chemistry* form the largest nodes, indicating their prominent influence in the field.

The overlay visualization highlights the average number of citations per paper across journals. The five most highly cited journals are *Food Chemistry* (1893 citations), *Foods* (469), *Molecules* (372), *Phytochemistry* (304), and *Sustainability* (287). The journals meeting the five-document threshold are also listed in Table 2. Collectively, the 162 papers published in these journals account for 22.5% of the total dataset (n = 720).

In terms of editorial representation, Elsevier (7 journals), MDPI (4 journals), and Wiley (3 journals) are the most prominent publishers. Notably, only 4 of the 20 journals are not indexed in the Journal Citation Reports (JCR), underscoring the predominance of recognized journals in the dataset. Furthermore, 14 of these journals are ranked within the top two JCR quartiles of their respective categories (7 in Q1 and 7 in Q2).

With respect to JCR classification, *Food Science and Technology* is the most frequently represented category, followed by disciplines related to Chemistry, Biochemistry, and Environmental Sciences (Table 2). These findings align with the conceptual analysis performed using VOSviewer (Figure 4).

## 4. Discussion

The world is currently facing major challenges, including rapid population growth and the global syndemic of obesity, undernutrition, and climate change. These issues underscore the urgent need to transform food systems toward sustainability to ensure the availability of high-quality foods, safeguard human health, extend healthy life expectancy, and protect the environment for the next generations [22,23].

Sweet potato is a resilient and adaptable crop with strong potential to enhance human nutrition due to its high content of essential nutrients and bioactive compounds. In a sustainability-oriented context, the valorization of sweet potato by-products (such as leaves, stems, stalks, and peels) depends on their specific composition, which can vary widely depending on factors such as cultivar type, soil characteristics, agronomic practices, and environmental conditions [24,25]. This variability underscores the importance of integrating agronomic, biochemical, and technological perspectives to optimize valorization strategies, a point that has been highlighted in recent studies on functional food development and circular economy approaches [6,18,26,27].

The SDGs were established by the United Nations in 2015, with a 15-year horizon for achievement, set in 2030. As this deadline approaches, robust analytical tools are needed to evaluate scientific progress toward these global goals. In this context, bibliometric methods are suited to manage large volumes of scientific data, revealing patterns, trends, collaboration networks, and offering a systematic, evidence-based approach to assess how research on food system sustainability contributes to advancing these objectives. By combining quantitative indicators (e.g., publication and citation metrics) with science-mapping techniques, bibliometric approaches enable researchers to track the evolution of knowledge, identify influential works, and detect emerging topics [28]. This study contributes innovatively by applying bibliometric tools not only to assess scientific productivity but also to connect sweet potato research with sustainability science, thereby bridging food chemistry, agricultural resilience, and industrial innovation.

The present bibliometric analysis shows a clear expansion of international research on sweet potato and sustainability during the past decade, reflecting the growing relevance and consolidation of this field. This expansion may reflect broader global trends in plant-based research and functional food innovation [29], reinforcing the role of sweet potato as a model crop for sustainable transitions. This trend may be associated with greater alignment with international policy agendas and the strengthening of collaborative research networks, highlighting its potential to generate meaningful benefits for society.

The keyword co-occurrence analysis indicates that the central terms *sweet potato* and *Ipomoea batatas* are frequently linked with topics related to its phytochemical compounds, health benefits, sustainable cultivation, and industrial applications. This pattern reveals a growing interest in connecting the biochemical composition of sweet potato with innovation and sustainability. Complementarily, the concepts co-occurrence analysis identified *Biology*, *Chemistry*, and *Food Science* as the most prominent and strongly interconnected disciplinary areas, which highlights the multidisciplinary nature of this research field. It is important to note that these clusters reflect the actual research landscape retrieved through our systematic search strategy, which explicitly included sustainability-related terms such as *biorefinery*, *sustainability*, *valorization*, *food waste*, and *circular economy*. In the keyword co-occurrence analysis, based on author-defined terms, *sustainability* emerges as one of the main nodes, indicating its explicit presence in the discourse of the publications. By contrast, in the concept co-occurrence analysis, where VOSviewer generates terms algorithmically, *sustainability* does not appear as a principal node. This distinction underscores that, although sustainability is present and recognized by authors, it is not yet dominant in the automatically derived conceptual structure of the field.

Those findings illustrate how sweet potato research is being aligned with sustainability principles through complementary approaches such as biochemistry, agriculture sustainability, industrial innovation, and biotechnology, shaping a comprehensive landscape of its scientific and socioeconomic relevance. Emerging clustered terms such as *biotechnology*, *genes*, and *genetics* highlight the importance of gene-editing techniques for genetic improvement, aimed at developing more resilient and productive sweet potato varieties [30,31]. This emphasis on biotechnology reflects a frontier of innovation, where genomic tools intersect with sustainability goals, offering practical pathways to enhance crop resilience and nutritional quality [32,33].

Despite the accelerated growth of scientific publications over the past decade, the co-authorship analysis reveals a certain degree of fragmentation and author isolation. Although fragmentation has not been quantified in this study, metrics such as network density, average clustering coefficient, or modularity index could provide more robust measures in future analyses [34,35]. Strengthening international scientific collaboration could not only further increase research output but also provide a more effective means of addressing global challenges related to food system sustainability. Such collaboration may be enhanced through targeted funding mechanisms, expanded mobility opportunities, the development of efficient digital platforms and tools, and the establishment of clear communication protocols and conflict resolution mechanisms. Our findings highlight that collaboration remains uneven, with structural inequalities that may reflect differences in visibility and impact, echoing concerns raised in global bibliometric studies on agricultural innovation [36,37]. In this context, the most productive authors identified in the bibliometric analysis illustrate different but complementary research directions: L.F. Trierweiler and J.L. Trierweiler focus on chemical engineering approaches related to bioprocesses and biorefinery of sweet potato, emphasizing process modeling, optimization, and sustainability assessment [38,39]. Meanwhile, A.P. Barros Júnior concentrates on an integrated approach that combines agronomy, food chemistry, and postharvest technology to enhance the nutritional value, functional properties, and industrial potential of sweet potato as a sustainable food crop [40,41].

The leading role of China as the primary producer of sweet potato in the world is reflected in the size of its node and its diversified partnerships. However, records of Chinese researchers receive a lower average number of citations compared to those from the United States. The United States has long occupied a central position in global research networks, supported by strong institutional visibility and widespread dissemination of research results. Moreover, American institutions frequently lead or take part in international collaborations, increasing the likelihood of cross-citations. While China has significantly expanded its research output in recent years, citation patterns may be associated with disparities in integration within citation schemes. This imbalance illustrates how scientific influence is not solely determined by production volume but by integration into global networks, a finding consistent with bibliometric analyses in other staple crops [42].

The growing participation of African countries such as Nigeria, Kenya, Uganda, Ghana, and Egypt, along with non-African nations as Malaysia or Papua New Guinea, highlights the importance of sweet potato crops in these regions, revealing the importance of research to boost local production systems and development strategies. Notably, countries such as the Netherlands, Italy, Iran, Denmark, and Germany, despite producing fewer publications, exhibit higher average citation rates than more prolific contributors such as China or India. This suggests that their scientific output, while being quantitatively smaller, holds greater influence and prestige in the field. Overall, the network structure also reveals that quantitative and qualitative indicators are not always correlated, and that scientific visibility and impact may be shaped less by agricultural relevance than by structural inequalities in research capacity, funding, and international cooperation. This observation provides a critical implication: strengthening equitable collaboration and capacity building in underrepresented regions is essential to ensure that sweet potato research contributes effectively to global sustainability agendas [37].

The journals with the greatest influence in this field were *Food Chemistry*, *Foods*, *Molecules*, *Phytochemistry*, and *Sustainability*, which reflects the central role of food science, chemistry, and sustainability in shaping current research trajectories. Furthermore, it is interesting that three out of five belong to MDPI, which is fully open access.

Scientific journals play a relevant role in the diffusion of knowledge. Core food-related journals such as *Food Chemistry*, *Foods*, and *Journal of Food Science* act as central hubs that consolidate methodologies and establish disciplinary standards, while more peripheral and interdisciplinary journals like *Sustainability*, *Journal of Environmental Management*, *Tropical Journal of Natural Products*, *Phytochemistry*, and *PLoS One* serve as bridges to translate technical results into broader contexts related to sustainability, policy, and society. This dual structure shows that journals shape the pathways through which research gains visibility, influence, and practical relevance, underscoring the importance of balancing disciplinary rigor with interdisciplinary dissemination to maximize scientific impact.

While this bibliometric study provides a broad and detailed overview of international scientific literature on sweet potato, several considerations should be considered when interpreting the findings. First, although the search strategy was systematic and based on three major databases (Web of Science Core Collection, Scopus, and PubMed), differences in coverage and indexing practices may have led to the omission of some relevant publications. Second, the focus on studies published in English introduces a language bias, which may have led to a partial underrepresentation of research from regions where sweet potato is widely cultivated but where English is not the primary language of scientific communication. However, English was chosen as the primary language of search because it remains the dominant medium of international scientific communication and ensures comparability across major scientific databases. Third, bibliometric indicators such as citation counts and co-authorship networks reflect patterns of scientific visibility rather than the intrinsic quality or local relevance of the research, which means that structural inequalities in funding, institutional capacity, and international collaboration may influence the results. Finally, the use of author keywords and database assigned terms may not fully capture emerging concepts or interdisciplinary nuances, particularly in rapidly evolving areas such as circular bioeconomy and by-product valorization. Despite these limitations, the study makes an innovative contribution by consolidating fragmented evidence into a coherent framework, offering both theoretical insights into the evolution of sweet potato research and practical implications for advancing equitable collaboration, sustainable innovation, and resilient food systems.

## 5. Conclusions

This bibliometric analysis shows that research on sweet potato has expanded rapidly in recent years, driven by growing interest in its nutritional value, bioactive compounds, and potential contributions to sustainable food systems. The thematic structure of the field reveals strong connections between biology, chemistry, and food science, while collaboration patterns highlight both established international networks and persistent fragmentation, particularly among countries that are major producers but have lower scientific visibility. The concentration of highly cited work in high income countries, many of which are not primary sweet potato producers, points to structural inequalities in research capacity and global knowledge production.

Future research should prioritize stronger integration between producing and non-producing countries, with an emphasis on equitable scientific collaboration and capacity building. There is also a need for deeper exploration of sweet potato within circular economy strategies, including the valorization of by-products, the development of sustainable processing technologies, and the characterization of bioactive compounds with potential industrial applications. Strengthening interdisciplinary approaches that connect agronomy, food science, biotechnology, and environmental sustainability will be essential for advancing the role of sweet potato in resilient and health promoting food systems. Finally, fostering open data practices, shared analytical platforms, and South–North research partnerships can help reduce existing disparities and accelerate knowledge transfer, ensuring that scientific advances contribute more directly to the SDGs, particularly those related to food security, health, and sustainable production.

## Figures and Tables

**Table 1:** Search strategy in Web of Science, Scopus, and PubMed databases.

Database	Search Strategy
Web of Science	Search 1: (“Sweet potato” OR “Ipomoea batatas”) (*All Fields*) AND (“bioactive compounds” OR “phytochemistry”) (*All Fields*). Search 2: (“Sweet potato” OR “Ipomoea batatas”) (*All Fields*) AND (“biorefinery” OR “sustainability” OR “valorization” OR “food waste” OR “circular economy”) (*All Fields*) Search 3: (“Sweet potato” OR “Ipomoea batatas”) (*All Fields*) AND (“bioactive compounds” OR “phytochemistry”) (*All Fields*), AND (“biorefinery” OR “sustainability” OR “valorization” OR “food waste” OR “circular economy”) (*All Fields*)
Scopus	Search 1: (“Sweet potato” OR “Ipomoea batatas”) (*Article title, Abstract, Keywords*) AND (“bioactive compounds” OR “phytochemistry”) (*Article title, Abstract, Keywords*). Search 2: (“Sweet potato” OR “Ipomoea batatas”) (*Article title, Abstract, Keywords*) AND (“biorefinery” OR “sustainability” OR “valorization” OR “food waste” OR “circular economy”) (*Article title, Abstract, Keywords*). Search 3: (“Sweet potato” OR “Ipomoea batatas”) (*Article title, Abstract, Keywords*) AND (“bioactive compounds” OR “phytochemistry”) (*Article title, Abstract, Keywords*) AND (“biorefinery” OR “sustainability” OR “valorization” OR “food waste” OR “circular economy”) (*Article title, Abstract, Keywords*)
PubMed	Search 1: (“Sweet potato” OR “Ipomoea batatas”) (*All Fields*) AND (“bioactive compounds” OR “phytochemistry”) (*All Fields*). Search 2: (“Sweet potato” OR “Ipomoea batatas”) (*All Fields*) AND (“biorefinery” OR “sustainability” OR “valorization” OR “food waste” OR “circular economy”) (*All Fields*) Search 3: (“Sweet potato” OR “Ipomoea batatas”) (*All Fields*) AND (“bioactive compounds” OR “phytochemistry”) (*All Fields*) AND (“biorefinery” OR “sustainability” OR “valorization” OR “food waste” OR “circular economy”) (*All Fields*)

**Table 2:** Information of journals with at least five records in the dataset. ISO3 country codes: BEL: Belgium; CHE: Switzerland, GBR: United Kingdom, MYS: Malaysia, NGA: Nigeria; USA: United States of America. IF: impact factor.

Journal Name	Papers in the Dataset	Publisher	Indexed in JCR	JCR Quartile (IF 2024)	JCR Category
*Foods*	21	MDPI (CHE)	Yes	Q1 (5.1)	Food Science and Technology
*Food Chemistry*	17	Elsevier (GBR)	Yes	Q1 (9.8)	Food Science and Technology; Nutrition and Dietetics; Chemistry, Applied.
*IOP Conference Series Earth and Environmental Science*	12	IOP Publishing (GBR)	No		
*Journal of Food Processing and Preservation*	10	Wiley (USA)	Yes	Q3 (2.5)	Food Science and Technology
*Sustainability*	10	MDPI (CHE)	Yes	Q3 (3.3)	Environmental Sciences; Environmental Studies; Green and Sustainable Science and Technology
*Molecules*	8	MDPI (CHE)	Yes	Q2 (4.6)	Chemistry, Multidisciplinary; Biochemistry and Molecular Biology
*Food Research*	7	Food Research Publisher (MYS)	No		
*Journal of Food Science*	7	Wiley (USA)	Yes	Q2 (3.4)	Food Science and Technology
*Phytochemistry*	7	Elsevier (GBR)	Yes	Q2 (3.4)	Biochemistry and Molecular Biology; Plant Sciences
*Food Science and Nutrition*	6	Wiley (USA)	Yes	Q2 (3.8)	Food Science and Technology
*International Journal of Food Science and Technology*	6	Oxford Univ Press (GBR)	Yes	Q2 (3.1)	Food Science and Technology
*LWT-Food Science and Technology*	6	Elsevier (GBR)	Yes	Q1 (6.6)	Food Science and Technology
*International Journal of Biological Macromolecules*	6	Elsevier (GBR)	Yes	Q1 (8.5)	Polymer Science; Chemistry, Applied; Biochemistry and Molecular Biology
*Tropical Journal of Natural Product Research*	6	Natural Product Research Group, University of Benin (NGA)	No		
*Journal of Agriculture and Food Research*	5	Elsevier (GBR)	Yes	Q1 (6.2)	Food Science and Technology; Agriculture, Multidisciplinary
*Food Bioscience*	5	Elsevier (GBR)	Yes	Q1 (5.9)	Food Science and Technology
*Applied Sciences*	5	MDPI (CHE)	Yes	Q2 (2.5)	Engineering, Multidisciplinary; Chemistry, Multidisciplinary; Physics, Applied; Material Science, Multidisciplinary
*Acta Horticulturae*	5	International Society for Horticultural Science (BEL)	No		
*Journal of Environmental Management*	5	Elsevier (GBR)	Yes	Q1 (8.4)	Environmental Sciences
*Plos One*	5	Public Library Science (USA)	Yes	Q2 (2.6)	Multidisciplinary Sciences

## Data Availability

Dataset is publicly available in the University of Chile Research Data Repository: https://datos.uchile.cl/dataset.xhtml?persistentId=doi:10.34691/UCHILE/HRVMA3 (accessed on 9 March 2026).

## Supplementary Materials

The following supporting information can be downloaded at: https://www.mdpi.com/article/10.3390/foods15061002/s1, Figure S1: Search strategy in the Web of Science database. Figure S2: Search strategy in Scopus database. Figure S3: Search strategy in PubMed database.
