# Supplementary material for: Mapping Sweet Potato Global Research for Sustainable Food Systems: A Bibliometric Perspective
Source: Foods. 2026 Mar 12;15(6):1002. doi: 10.3390/foods15061002 (PMC13025605; doi:10.3390/foods15061002)
Supplement: Supplementary file 1 [file foods-15-01002-s001.zip › foods-4158542-supplementary.pdf]

**SUPPLEMENTARY MATERIAL**

**Paper: Mapping sweet potato global research for sustainable food systems: a bibliometric perspective.**

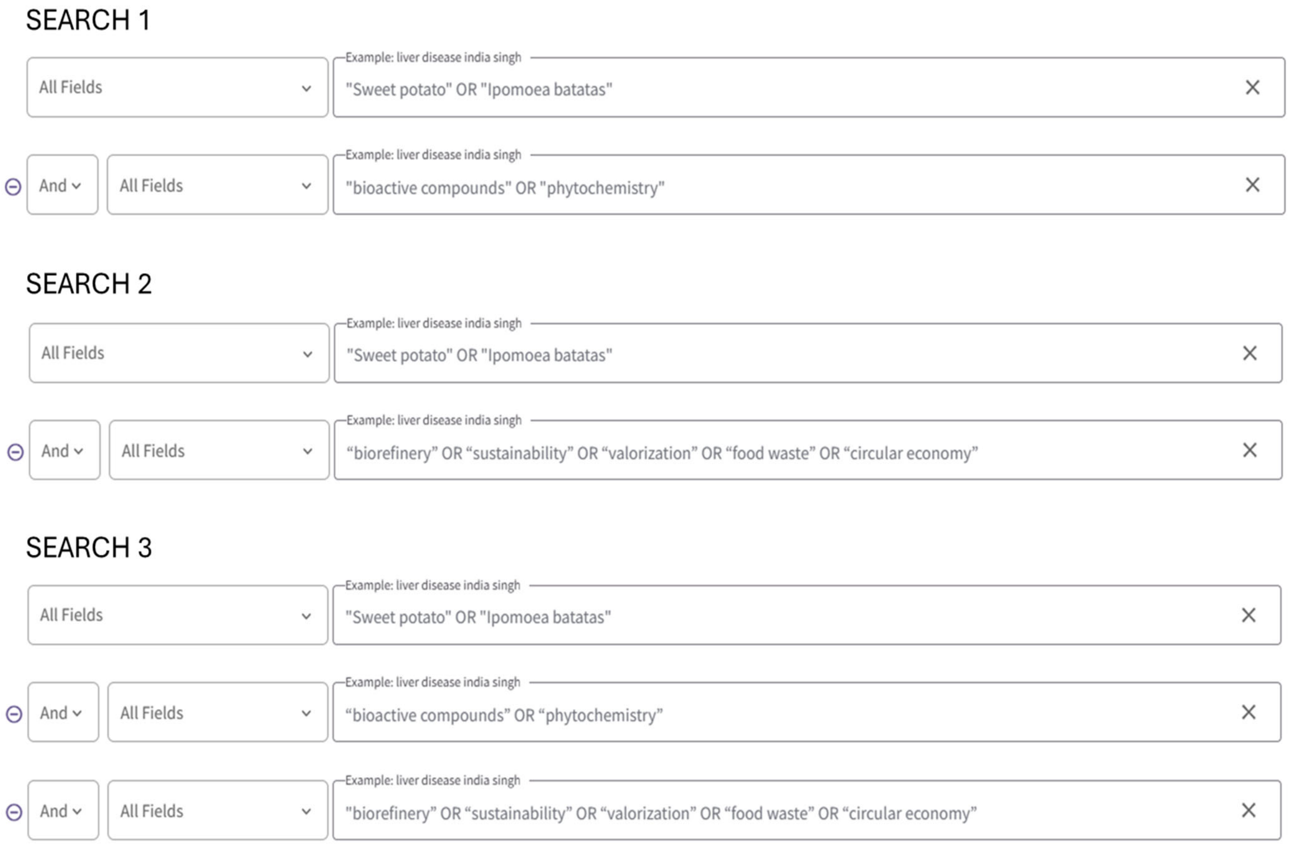


Supplementary Figure S1. Search strategy in the Web of Science database.

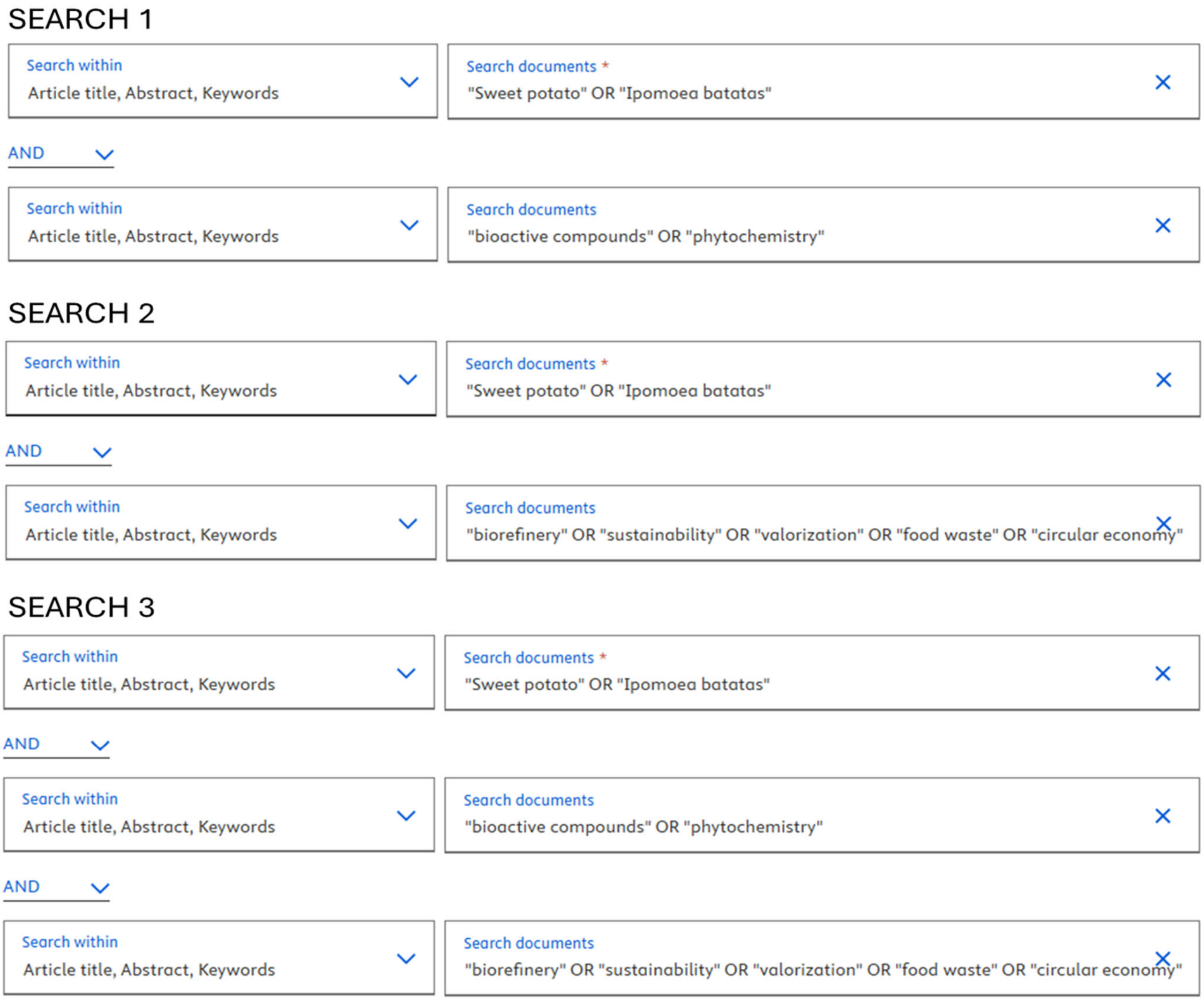


Supplementary Figure S2. Search strategy in Scopus database.

## SEARCH 1

Query box

("Sweet potato" OR "Ipomoea batatas") AND ("bioactive compounds" OR "phytochemistry")

## SEARCH 2

Query box

("Sweet potato" OR "Ipomoea batatas") AND ("biorefinery" OR "sustainability" OR "valorization" OR "food waste" OR "circular economy")

## SEARCH 3

Query box

("Sweet potato" OR "Ipomoea batatas") AND ("bioactive compounds" OR "phytochemistry") AND ("biorefinery" OR "sustainability" OR "valorization" OR "food waste" OR "circular economy")

Supplementary Figure S3. Search strategy in PubMed database.
